# Flexible Carbon Nanotube-Based Polymer Electrode for Long-Term Electrocardiographic Recording

**DOI:** 10.3390/ma12060971

**Published:** 2019-03-23

**Authors:** Miao Chi, Jingjing Zhao, Ying Dong, Xiaohao Wang

**Affiliations:** 1 Graduate School at Shenzhen, Tsinghua University, University Town of Shenzhen, Shenzhen 518055, China; chim16@mails.tsinghua.edu.cn (M.C.); wang.xiaohao@sz.tsinghua.edu.cn (X.W.); 2 Tsinghua-Berkeley Shenzhen Institute, Tsinghua University, University Town of Shenzhen, Shenzhen 518055, China; jjzhao2017@sz.tsinghua.edu.cn

**Keywords:** ECG electrode, long-term monitoring, carbon nanotube, flexible composite electrode

## Abstract

The long-term monitoring of electrocardiogram (ECG) is critical for the accurate diagnosis and tracking of cardiovascular diseases (CVDs). However, the commercial Ag/AgCl electrode is not suitable for long-term monitoring due to skin allergies and signal degradation, caused by the conductive gel drying over time. In this paper, a flexible gel-free electrode, composed of a multi-wall carbon nanotube (MWCNT) and polydimethylsiloxane (PDMS), is proposed for long-term wearable ECG monitoring. To achieve uniform dispersion of MWCNTs in viscous PDMS, we developed a novel parallel solvent-assisted ultrasonic dispersion method, wherein the organic solvent n–Hexane served as a dispersion to avoid MWCNT aggregates. The properties of the MWCNT/PDMS electrode were assessed through structural characterization, contact impedance tests, ECG measurements, and biocompatibility tests. When the MWCNT weight fraction reached 5.5 wt%, the skin-electrode contact impedance of the MWCNT/PDMS electrode was lower than that of the Ag/AgCl electrode below 100 Hz. In daily ECG monitoring, the MWCNT/PDMS electrodes showed superior performance against motion artifact compared to the Ag/AgCl electrode. After seven days of wearing the MWCNT/PDMS electrode, ECG signals did not degrade and no side effects, such as skin redness and swelling, were observed. Thus, this electrode could enable long-term ECG monitoring in wearable healthcare systems.

## 1. Introduction

Cardiovascular diseases (CVDs), as reported by the World Health Statistics 2017, issued by the World Health Organization (WHO), are the greatest threats to human health [1]. At present, the accurate diagnosis and development track of CVDs are mainly based on electrocardiogram (ECG) measurement which records the electrical potential differences on body surfaces during each cardiac cycle. The most commonly used electrode for electrophysiological signal acquisition is the silver/silver chloride (Ag/AgCl) electrode with conductive paste. With the fast development of wearable medical devices for health monitoring, ECG measurement has evolved from one-time hospital examination to long-term home care. In this situation, the conventional Ag/AgCl electrode is not suitable for long-term monitoring due to skin allergies and signal degradation, caused by the conductive gel drying over time [2]. To resolve these problems, gel-free electrodes have been intensively investigated.

So far, all dry surface electrodes can be categorized into three types: capacitive electrodes, penetrating electrodes, and surface electrodes. A capacitive electrode is based on the capacitive coupling principle to collect ECG signals [3]. It does not come into direct contact with skin and is normally isolated with clothing, air, or other insulating material. Walter Reed Army Institute of Research carried out ECG measurement on soldiers by integrating capacitive electrodes on a chest strap [4]. Furthermore, ECG signals have been acquired through the “Aachen Smart Chair” [5], a bathtub [6], or wireless sensor networks [7,8]. However, with capacitive electrodes, the ECG signal is more sensitive to circuit noise and is severely influenced by motion artifacts. The penetrating electrodes, which have sharp micro-structured surfaces, obtain ECG signals by piercing into the insulating stratum corneum that consists of dead cells. Micrometer-sharp silicon structures, such as microneedle array [9,10,11,12], barbed microtips [13], and micromachined spikes [14] have been designed to achieve lower skin-electrode contact impedance compared to the commercial wet electrode. Although penetrating electrodes effectively reduce motion artifact and contact impedance, this kind of dry electrode cannot be used for long-term ECG recording due to bad properties in mechanical stability and wearing comfort. The surface electrode is formed by flexible polymer and conductive nanomaterial. The nanocomposite electrodes reveal good performance in wearability and motion artifact resistance due to their excellent conformability to skin. Metallic nanomaterials [15], such as silver nanoparticles [16], silver nanowire [17,18], and nickel powder [19] have been employed as conductive filler. However, the high contact impedance of metallic nanocomposite limits its practical use for accurate ECG recording.

In this paper, we present a multi-wall carbon nanotube (MWCNT)/polydimethylsiloxane (PDMS) composite electrode which is a great candidate for long-term wearable ECG monitoring with great robustness. PDMS has been widely applied in biomedical sensors [20,21,22] for its mechanical flexibility, biocompatibility, and amenability of molding. In addition, MWCNT is the most appropriate and attractive filler for a polymer-based electrode, because it has high respect ratio, high conductivity, and is easily tangled with each other to form the 3-D conductive network within the polymer matrix [23]. Thus, the MWCNT/PDMS electrode, which combines excellent properties of these two attractive materials, can be a promising candidate in long-term ECG monitoring. Here, we propose a parallel solvent-assisted ultrasonic dispersion method to address the problem of MWCNT aggregates. The Fourier transform infrared spectroscopy (FT-IR) and Raman spectroscopy were used to characterize the structure of the MWCNT/PDMS composite. To determine the optimal MWCNT concentration of the conductive composite, its electrical property was evaluated through a contact impedance experiment. The ECG signal was measured under resting and walking using MWCNT/PDMS electrodes with different concentrations. To investigate the performance of MWCNT/PDMS electrodes in long-term service, the ECG signals of different MWCNT concentrations were recorded on 0, 2, 5, 7 days since wearing electrodes, and the cytotoxicity and skin compatibility tests were designed to examine the biocompatibility of MWCNT/PDMS electrodes.

## 2. Materials and Methods

### 2.1. MWCNT/PDMS Composite Preparation

**Figure 1:**
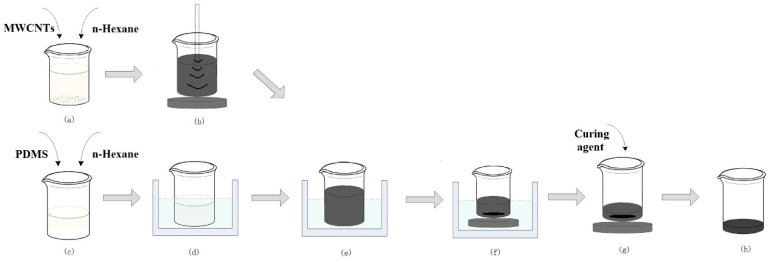
Preparation process flow of the multi-wall carbon nanotube (MWCNT)/polydimethylsiloxane (PDMS) composite. (**a**) MWCNTs and n-Hexane were mixed in a mass ratio of 1:200. (**b**) MWCNTs were dispersed in n-Hexane through tip sonication. (**c**) PDMS and n-Hexane were mixed in a mass ratio of 1:5. (**d**) PDMS was dispersed in n-Hexane through a bath sonicator. (**e**) The MWCNTs dispersion was added into the PDMS dispersion and processed continuously in the bath sonicator. (**f**) The mixture was placed in a water bath at 75 °C under magnetic stirring until the n-Hexane was totally volatilized. (**g**) The curing agent (mass ratio to PDMS 1:10) was added into the mixture made from MWCNT/PDMS and then magnetically stirred. (**h**) The homogeneous MWCNT/PDMS composite was ready for molding.

During the preparation of the conductive nanocomposite, MWCNTs are easy to form aggregates because of the strong van der Waals interaction. To achieve uniform dispersion of MWCNTs in viscous PDMS, a parallel solvent-assisted ultrasonic dispersion method (Figure 1) was developed. The procedure is as follows. Firstly, MWCNTs (ID: 3–5 nm, OD: 8–15 nm, length: 50 μm, purity > 95%, Aladdin Bio-Chem Technology Co., LTD., Shanghai, China) were dispersed in n-Hexane ( Analytical Reagent, 97%, Aladdin Bio-Chem Technology Co., LTD., Shanghai, China) at 0.5 wt% through tip sonication (Power: 360 W, JY92–IIN, Scientz Biotechnology Co., LTD., Ningbo, China) for 90 min (Figure 1a,b). Simultaneously, PDMS (Sylgard184, Dow Corning Company, Midland, MI, USA) was dispersed in n-Hexane at 20 wt% through a bath sonicator (Power: 120 W, Yunyi Technology Co., Ltd., Shenzhen, China) for 30 min (Figure 1c,d). Subsequently, the MWCNTs dispersion was added into the PDMS dispersion and processed continuously in the bath sonicator for 5 hours (Figure 1e). Then the mixture was placed in a water bath at 75 °C under magnetic stirring until the n-Hexane was totally volatilized (Figure 1f). Eventually, the curing agent (mass ratio to PDMS 1:10) was added into the mixture made from MWCNT/PDMS and then magnetically stirred at room temperature for 5 min (Figure 1g,h). 

### 2.2. Fabrication of the MWCNT/PDMS Electrode

**Figure 2:**
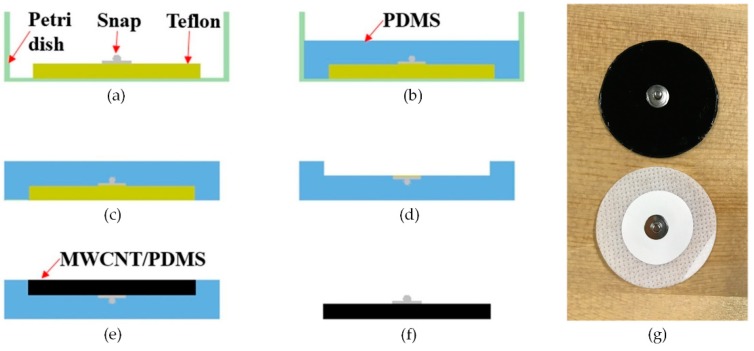
Fabrication process of the MWCNT/PDMS electrode. (**a**) A circular Teflon substrate and a metal snap were placed on petri dish. (**b**) The uniform mixture of the PDMS precursor and crosslinking agent was poured and cured in a vacuum oven. (**c**) The PDMS master mold was detached from the petri dish. (**d**) The Teflon was removed from the PDMS mold. (**e**) The prepared homogeneous mixture of MWCNTs and PDMS was poured into the PDMS mold and thermally cured in a vacuum oven. (**f**) A flexible dry MWCNT/PDMS electrode was detached from the PDMS mold. (**g**) The fabricated MWCNT/PDMS electrode (top) and the commercial Ag/AgCl electrode (bottom).

Figure 2a–f shows a schematic diagram of the fabrication process of the MWCNT/PDMS electrode. The fabricated MWCNT/PDMS electrode (top) and the commercial Ag/AgCl electrode (bottom) (Huaxi Medical Equipment Co., Ltd., Xinxiang, China) are illustrated in Figure 2g. As is shown, a metal snap (material: Copper, surface treatment: Nickel plating, diameter: 10 mm), which could be straightforwardly connected to the conventional ECG cable with a clip, was placed on a Teflon substrate with a diameter of 50 mm (Figure 2a). Following this, the uniform mixture of the PDMS precursor and crosslinking agent was poured and cured in a vacuum oven (Bluepard Instruments Co., Ltd., Shanghai, China) at 80 °C (Figure 2b). After 1 hour, the PDMS master mold was detached from the petri dish (Figure 2c), and the Teflon was carefully removed from the PDMS mold (Figure 2d). Subsequently, the prepared homogeneous mixture of MWCNTs and PDMS was poured into the PDMS mold and thermally cured in a vacuum oven at 70 °C for 3 hours (Figure 2e). Finally, a flexible dry MWCNT/PDMS electrode was detached from the PDMS mold (Figure 2f). 

In order to investigate the influence of MWCNT concentration on the contact impedance of the nanocomposite electrode, a series of electrodes with different weight fracture of MWCNT were fabricated to determine the optimal composition proportion.

## 3. Results and Discussion

### 3.1. Structural Characterization

The FT-IR and Raman spectroscopy were used to characterize the structure of the MWCNT/PDMS composite (MWCNT concentration: 4 wt%). 

**Figure 3:**
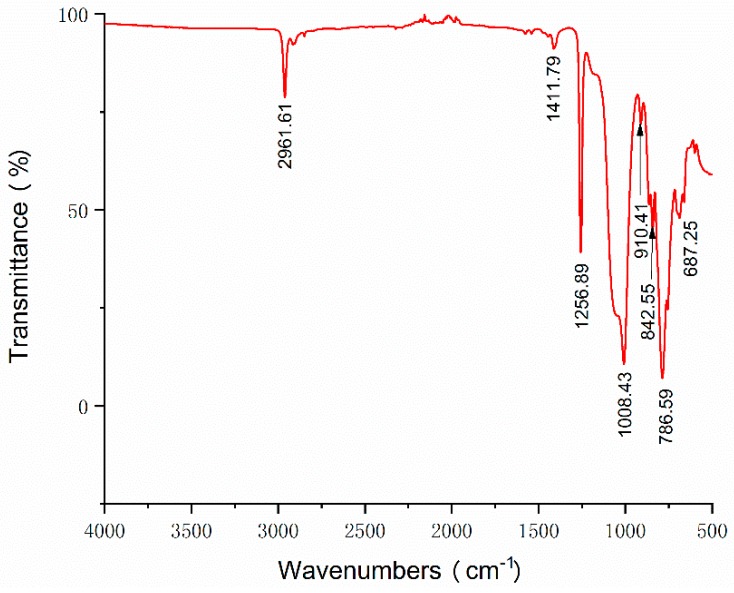
FT-IR spectra of the MWCNT/PDMS composite (MWCNT concentration 4 wt%).

The FT-IR spectra of the MWCNT/PDMS composite were recorded by a FT-IR spectrometer (Thermo Scientific Nicolet iS 50, Thermo Fisher Scientific Co., Ltd., Waltham, MA, USA) within the range of 500–4000^−1^. The test sample was mixed with KBr pellets (MWCNT/PDMS composite to KBr, 1:150). The distinctive peaks of the FT-IR spectra for the MWCNT/PDMS composite are digitally labeled in Figure 3. 

The absorption band at 2961 cm^−1^ is corresponding to C–H stretching of CH_3_. The peak at 1411 cm^−1^ is considered as the C=C bending vibration of carbon nanotubes [24]. The Si–CH_3_ bands are observed at 1256 cm^−1^ and in 680–850 cm^−1^ regions [25], which illustrates the existence of the polysiloxane group in PDMS. The wide absorption band at 1008 cm^−1^ is associated to symmetrical Si–O–Si stretching. And the peak at 910 cm^−1^ is attributed to the asymmetrical Si–O–Si stretching in the composite [26], which indicates the success of the synthesis of the MWCNT/PDMS composite.

In order to further prove the synthesis of the MWCNT/PDMS composite, the Raman experiments were performed using a Raman spectrometer (LabRAM HR800, HORIBA Jobin Yvon Co., Ltd., Paris, France) with a 532 nm excitation wavelength. Each Raman spectrum of samples was obtained by 5 s exposures over three accumulations. 

**Figure 4:**
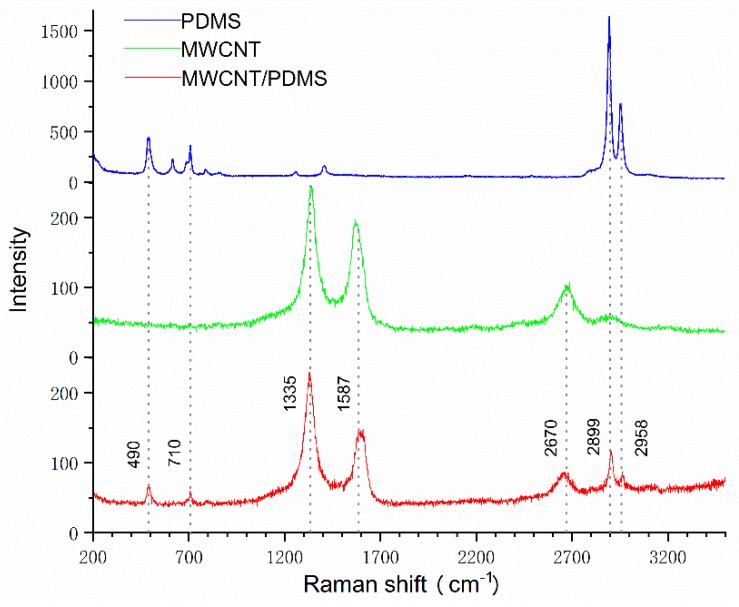
Raman spectra of the PDMS, MWCNTs, and the MWCNT/PDMS composite sheet.

Figure 4 illustrates the Raman spectra of the PDMS, MWCNT, and MWCNT/PDMS composite sheet. In the case of the PDMS sheet, the prominent Raman bands belong to Si–O–Si (490 cm^−1^), Si–C symmetric stretching (615 cm^−1^ and 710 cm^−1^), CH_3_ symmetric stretching (2899 cm^−1^), and CH_3_ asymmetric stretching (2958 cm^−1^) [27]. In the Raman spectra of MWCNT, the disorder band (D band) at 1335 cm^−1^, the graphite band (G band) at 1587 cm^−1,^ and the second order of the D band (2D band) at 2670 cm^−1^ are clearly observed [28]. The intensity ratio of D and G bands (I_D_/I_G_) is generally regarded as an indicator of defects on MWCNTs [29]. The calculated I_D_/I_G_ values from Raman spectra increase from 1.24 to 1.54 for the MWCNT and the MWCNT/PDMS respectively, which shows that the defects on MWCNT’s increase is owing to the sonication process during composite preparation. As is shown, the major Raman bands of the MWCNT/PDMS composite are exactly corresponding to the typical Raman bands of both PDMS and MWCNT, which manifests that the composite is synthesized by PDMS and MWCNT.

### 3.2. Skin-Electrode Contact Impedance Measurement

**Figure 5:**
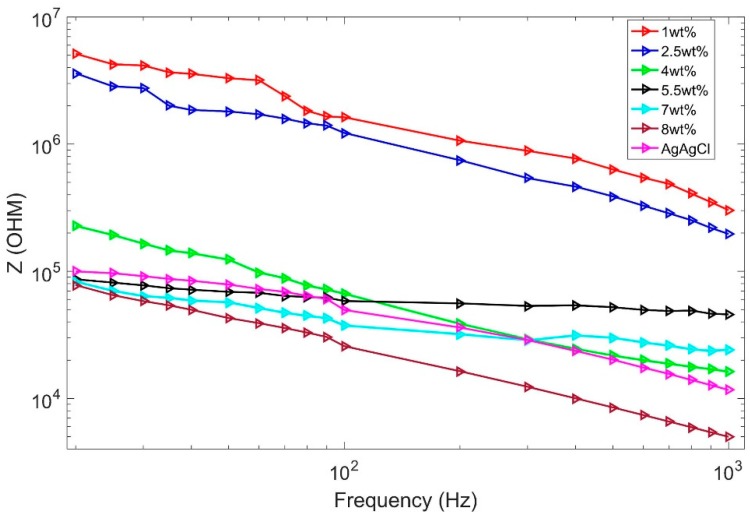
Skin–electrode contact impedance comparison.

The contact impedance between skin and electrode is a significant indicator to evaluate the electrical property of the ECG electrode. It could be easily influenced by skin moisture, pressure, and electrode size [30,31]. For reproducible results, the measurement conditions were set as follows: (1) MWCNT/PDMS electrodes were placed at the same locations on the forearm with a center distance of 10 cm, (2) there was no external force applied to electrodes, (3) sweat and dust on the skin was wiped off with an alcohol prep pad before each measurement, (4) the diameter and thickness of the MWCNT/PDMS electrodes were 50 mm and 500 μm according to the proposed fabrication process. With a LCR meter (TH2828S, Tonghui Electronics Co., Ltd., Changzhou, China), seven group tests were carried out on a healthy male volunteer aged 24 years old. The composition of MWCNT in PDMS varied from 1 wt% to 8 wt%, and the commercial Ag/AgCl electrode (diameter 50 mm) was used as a control experiment. We recorded the skin-electrode contact impedance according to frequency ranges of 20 Hz–1 kHz and plotted the logarithmic curve in Figure 5.

According to Figure 5, the skin-electrode contact impedance decreases as the concentration of MWCNT increases. When the MWCNT ratio reaches 5 wt%, the contact impedance of the composite electrode is lower than that of the commercial wet electrode. Moreover, the electrodes with a concentration ratio between 5.5 wt% and 8 wt% show little difference in contact impedance below 100 Hz, indicating that MWCNT ratio gradually becomes saturated within this range.

### 3.3. Short-Term ECG Measurement

**Figure 6:**
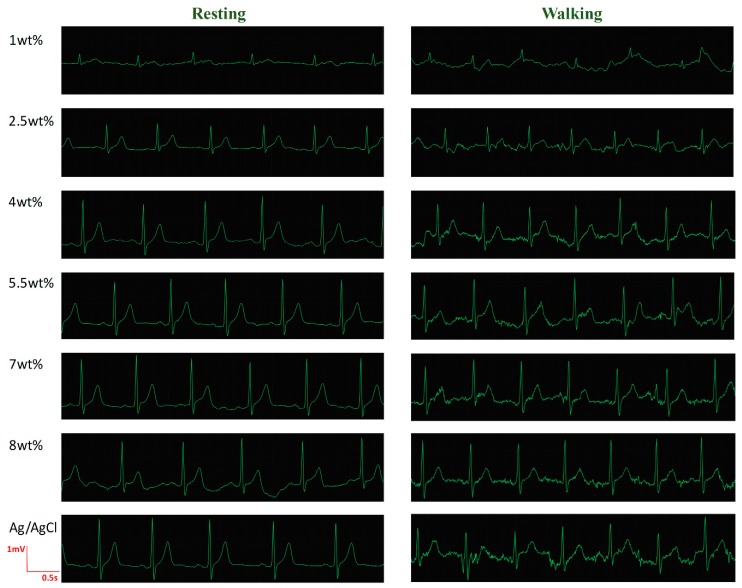
Electrocardiogram (ECG) signals of a male volunteer (age: 23) under resting and walking state for various MWCNT concentrations.

The ECG signals (Figures S1–S5) of five subjects were recorded using an ECG workstation (SE-1200 Express, Edan Instruments, Inc., Shenzhen, China). With the help of 3M type, MWCNT/PDMS electrodes and commercial Ag/AgCl electrodes (control experiment) were placed on lead V5. Three electrodes for each MWCNT concentration were tested to verify the stability of the electrode system. For reproducible results, the skin test area of the five subjects were cleaned by alcohol prep pads prior to each measurement. With the Wilson’s central terminal as a reference potential, the typical ECGs of a male volunteer under resting and walking state are shown in Figure 6.

The ECG of the 1 wt% MWCNT/PDMS electrode presents the lowest signal amplitude and the largest motion artifact. The important features of the ECG, such as P waves and T waves, are barely observed at 1 wt% concentration. Although the signal amplitude obtained by the 2 wt% MWCNT/PDMS electrode is still lower than that obtained by the Ag/AgCl electrode, the sensitivity to motion artifact is effectively reduced, and the T waves are clearly detected. As the weight fraction of MWCNT increases, the amplitude of the ECG signal gets higher. The signal amplitudes with MWCNT concentration from 4 wt%–8 wt% show no critical differences, which is consistent with the previous conclusion from the contact impedance test. The MWCNT/PDMS electrodes with concentration above 4 wt% provide comparable signal quality to the wet electrode under resting. The P, QRS, and T waves are clearly observed. Additionally, the Ag/AgCl electrode involves greater noise and baseline drift under walking, which demonstrates that the MWCNT/PDMS electrodes exhibit superior performance against the motion artifact compared to the Ag/AgCl electrode under motion. 

**Figure 7:**
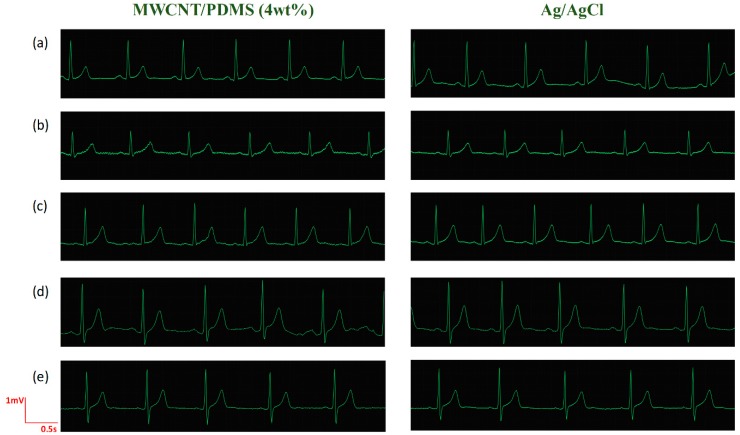
ECG signals of five subjects under resting state for 4 wt% MWCNT/PDMS electrodes and Ag/AgCl electrodes. Subjects: (**a**) male aged 38, (**b**) female aged 31, (**c**) female aged 24, (**d**) male aged 23, (**e**) male aged 18.

To investigate the variability in result for use of MWCNT/PDMS electrodes, the ECG signals of five subjects (three male and two female; age: 18–38) were measured under resting state using 4 wt% MWCNT/PDMS electrodes and Ag/AgCl electrodes. As shown in Figure 7, the ECG signal amplitude varies from person to person, which stems from the discrepancy in the skin-electrode contact impedance of different subjects. On the other hand, for each subject, the ECG signals obtained by MWCNT/PDMS electrodes are comparable to that obtained by commercial Ag/AgCl electrodes, which interprets that the MWCNT/PDMS electrodes could be applied to various users in ECG monitoring.

### 3.4. Long-Term ECG Measurement

**Figure 8:**
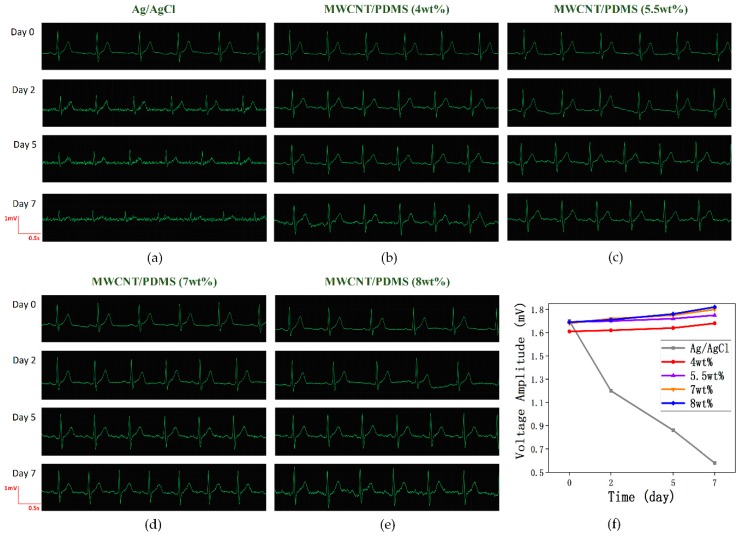
Comparison of long-term ECG detection with commercial Ag/AgCl electrodes and MWCNT/PDMS electrodes. ECG signals were measured using (**a**) commercial Ag/AgCl electrodes, (**b**) 4 wt%, (**c**) 5.5wt%, (**d**) 7 wt%, and (**e**) 8 wt% MWCNT/PDMS electrodes. (**f**) R-wave average amplitude as a function of time and 5 types of electrodes.

In order to examine the MWCNT/PDMS electrode performance for long-term use, the ECG signals were recorded on 0, 2, 5, 7 days since wearing electrodes. Three electrodes for each type were tested to ensure the reliability of the results. In resting state, the comparison of long-term ECG detection using commercial Ag/AgCl electrodes and MWCNT/PDMS electrodes (MWCNT concentration: 4 wt%, 5.5 wt%, 7 wt%, 8 wt%) is shown in Figure 8.

For the Ag/AgCl electrode, the reduction of ECG signal amplitude is evident after two days and the P waves, which is an important gist in cardiovascular disease diagnosis, are totally submerged in noise due to dehydration of the conductive gel over time. In addition, Figure 8f reveals that there has been a steep decrease for the R-wave average amplitude of ECG signals obtained by Ag/AgCl electrodes. In contrast, no significant changes in ECG signals obtained by MWCNT/PDMS electrodes are observed from day 0 to day 7. The signal amplitude slightly increases with time, which is caused by the accumulation of skin secretions, reducing the skin-electrode contact impedance. As discussed above, the MWCNT/PDMS electrodes do not degrade and show excellent characteristics in long-term ECG monitoring.

### 3.5. Cytotoxicity and Skin Compatibility Tests

**Figure 9:**
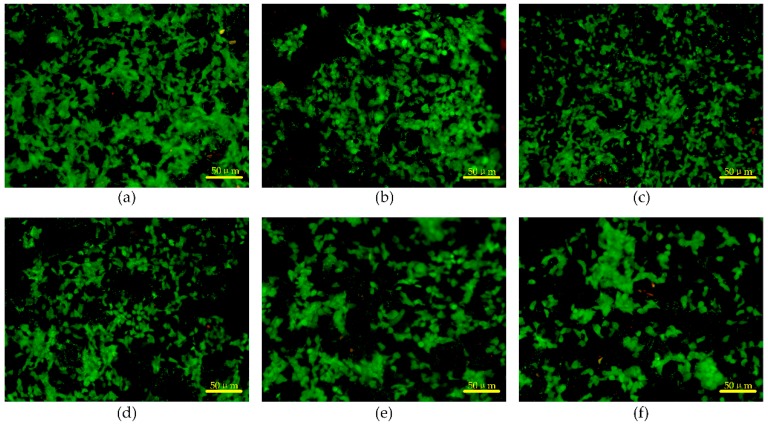
The result of cytotoxicity tests on (**a**) 1 wt%, (**b**) 2.5 wt%, (**c**) 4 wt%, (**d**) 5.5 wt%, (**e**) 7 wt%, and (**f**) 8 wt% MWCNT/PDMS composite sheets.

To test the cytotoxicity of the composite electrode, the immortal human keratinocyte line HaCaT cells were cultured on six types of MWCNT/PDMS composite sheets (MWCNT concentration: 1 wt%, 2.5 wt%, 4 wt%, 5.5 wt%, 7 wt%, 8 wt%) for seven days. The size of these MWCNT/PDMS sheets is 1 cm × 1 cm × 0.05 cm. The HaCaT cells were washed and detached using the phosphate buffered saline (Corning Inc., Corning, NY, USA) and Tryp-LE Express buffer (Invitrogen Corporation, Carlsbad, CA, USA), respectively. After counting with a hemocytometer, 1 × 10^4^ HaCaT cells were seeded on the surface of each MWCNT/PDMS sheet and cultured for seven days in Dulbecco’s modified Eagle medium (DMEM, Corning Inc., Corning, NY, USA) containing 10% fetal bovine serum and 100 μg/mL penicillin/streptomycin (Gibco Company, Waltham, MA, USA), in humidified 5% CO_2_ atmosphere at 37 °C. The viability of HaCaT cells was evaluated using a Live/Dead Assay Kit (Invitrogen). As shown in Figure 9, the live cells and the dead cells are respectively stained green and red. Most cells are alive and proliferate uniformly on the surface of MWCNT/PDMS sheets. The viability of HaCaT cells on all MWCNT/PDMS sheets surpasses 90%, demonstrating that the MWCNT/PDMS composite electrodes are nontoxic to cells.

**Figure 10:**
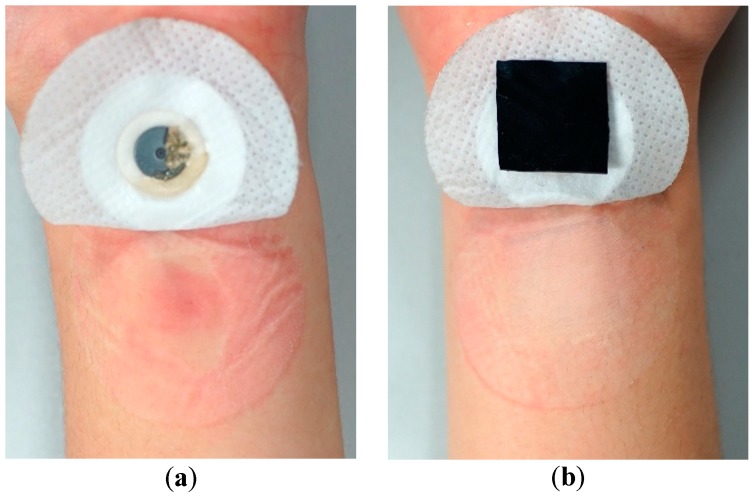
Skin reaction comparison of wearing an Ag/AgCl electrode (**a**) and a MWCNT/PDMS sheet (**b**) for 7 days.

We also carried out the skin compatibility test, wherein the commercial electrode and the MWCNT/PDMS composite sheet were attached to the same location of the forearm for seven days with an adhesive bandage. Figure 10 presents the results of skin reaction. Long-term use of the wet electrode caused severe side effects, such as skin redness and swelling, whereas the skin was found normal and no skin irritation was observed with the nanocomposite sheet. The result verifies that the MWCNT/PDMS electrode has good compatibility with skin in long-term service.

## 4. Conclusions

In this work, we fabricated a flexible dry electrode consisting of PDMS and MWCNT in a simple and efficient manner for long-term ECG monitoring. A parallel solvent-assisted ultrasonic dispersion method was adopted to achieve a homogeneous MWCNT/PDMS composite. The dry electrode was drop-casted in a master mold with a metal snap inside, which could be straightforwardly connected to a conventional ECG machine. The properties of the MWCNT/PDMS electrode were assessed through structural characterization, a contact impedance test, an ECG measurement, and biocompatibility tests. The success of the synthesis of the MWCNT/PDMS composite was approved by the FT-IR and Raman spectroscopy. When the weight fraction of MWCNT reached 5.5 wt%, the contact impedance of the dry electrode was lower than that of the Ag/AgCl electrode below 100 Hz. Moreover, the ECG signals obtained by the proposed dry electrode showed better performance under motion compared to the Ag/AgCl electrode. The cytotoxicity and skin compatibility test indicated that the nanocomposite electrode was nontoxic to cells and did not cause noticeable skin irritation, such as redness or swelling. In contrast to commercial wet electrodes, no significant degradation of ECG signal quality was observed after seven days of use. Additionally, dry electrodes in the market are mostly made by hard metal materials, introducing motion artifacts and high contact impedance due to the unconformability with wrinkled skin. However, the MWCNT/PDMS electrode is flexible and conformal with skin, showing superior performance against the motion artifact. The aforementioned results demonstrate that the MWCNT/PDMS electrode can overcome the limitations of commercial wet electrodes and dry electrodes due to its robust performance and skin biocompatibility. We expect the proposed MWCNT/PDMS electrodes will be used in wearable healthcare systems and contribute a lot to long-term ECG monitoring.

## Supplementary Materials

The following are available online at https://www.mdpi.com/1996-1944/12/6/971/s1, Figure S1: ECG signals of a male volunteer (age: 38) under resting and walking state for various MWCNT concentrations, Figure S2: ECG signals of a female volunteer (age: 31) under resting and walking state for various MWCNT concentrations, Figure S3: ECG signals of a male volunteer (age: 24) under resting and walking state for various MWCNT concentrations, Figure S4: ECG signals of a male volunteer (age: 23) under resting and walking state for various MWCNT concentrations, Figure S5: ECG signals of a male volunteer (age: 18) under resting and walking state for various MWCNT concentrations.
