# Supplementary material for: Flexible Carbon Nanotube-Based Polymer Electrode for Long-Term Electrocardiographic Recording
Source: Materials (Basel). 2019 Mar 23;12(6):971. doi: 10.3390/ma12060971 (PMC6470748; doi:10.3390/ma12060971)
Supplement: Supplementary file 1 [file materials-12-00971-s001.pdf]

Supplementary Information for

Flexible Carbon Nanotube-Based Polymer Electrode for Long-Term Electrocardiographic Recording

Miao Chi, Jingjing Zhao, Ying Dong, Xiaohao Wang

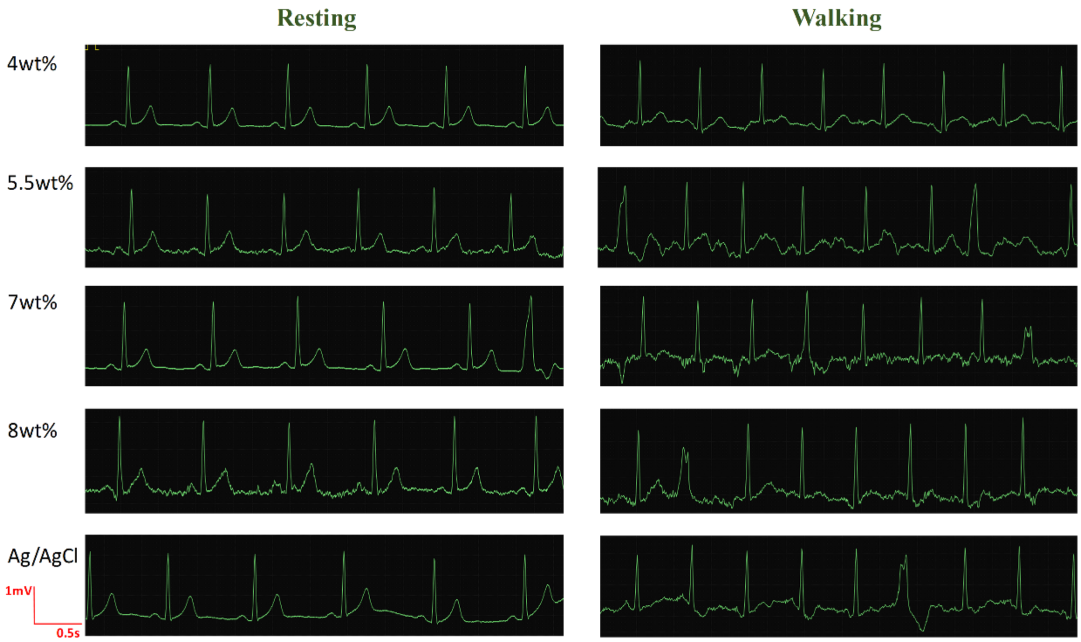

Figure S1. ECG signals of a male volunteer (age: 38) under resting and walking state for various MWCNT concentrations.

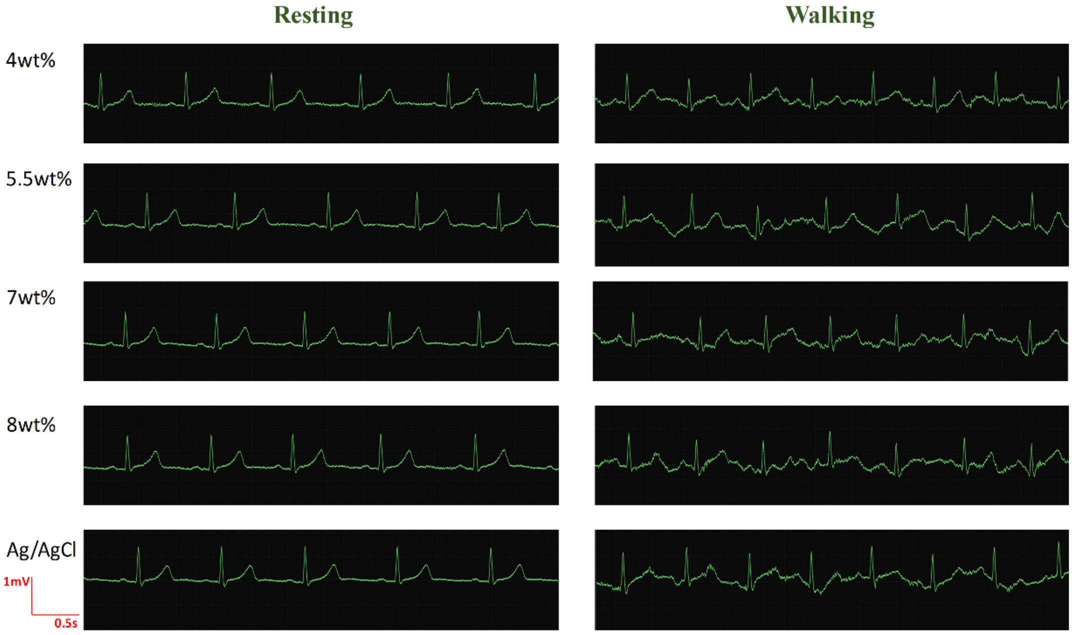

Figure S2. ECG signals of a female volunteer (age: 31) under resting and walking state for

various MWCNT concentrations.

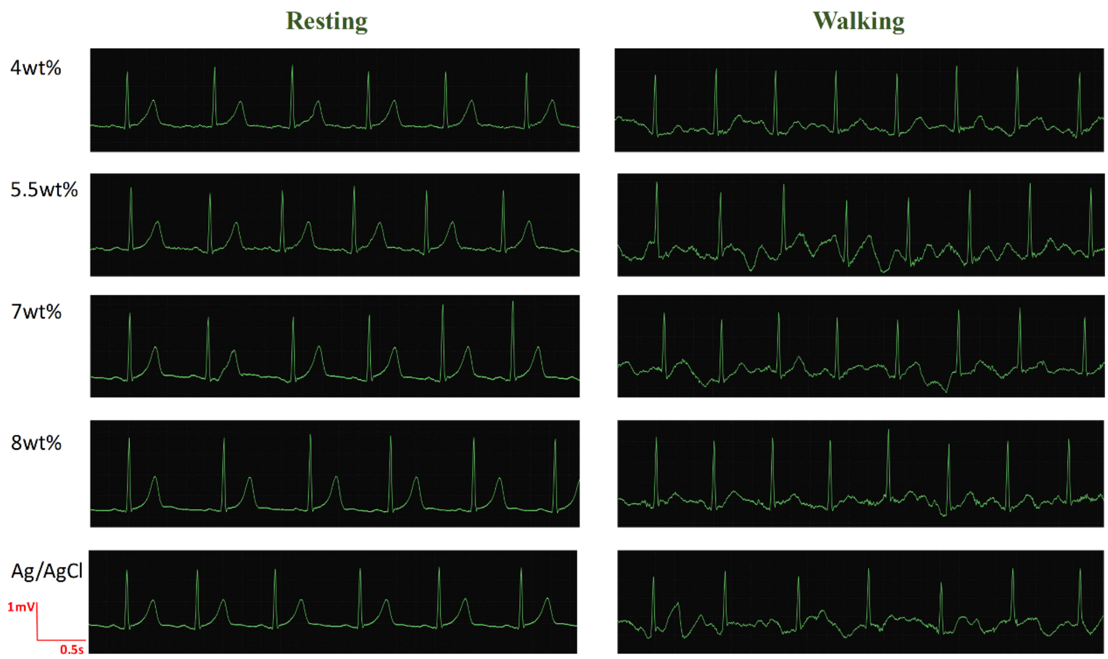

Figure S3. ECG signals of a female volunteer (age: 24) under resting and walking state for various MWCNT concentrations.

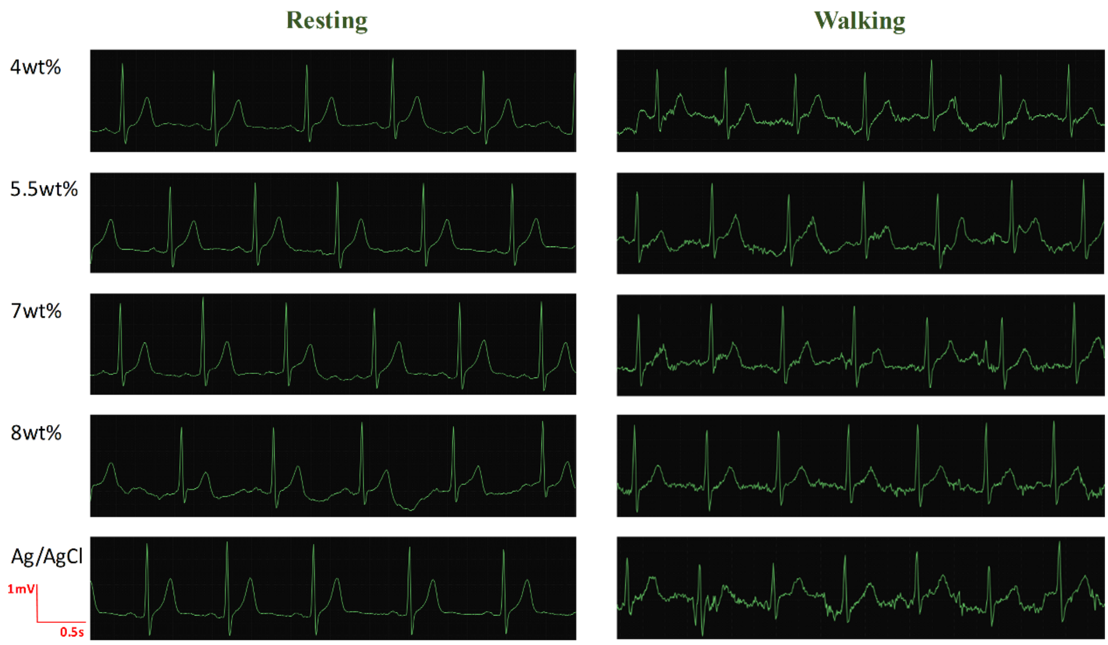

Figure S4. ECG signals of a male volunteer (age: 23) under resting and walking state for various MWCNT concentrations.

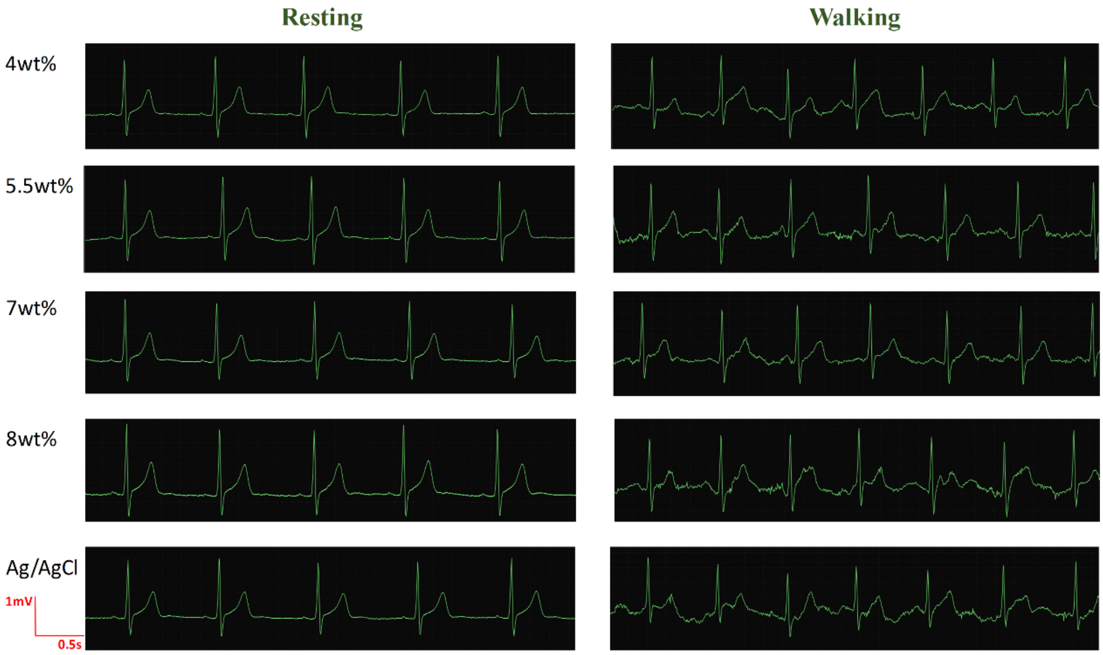

Figure S5. ECG signals of a male volunteer (age: 18) under resting and walking state for various MWCNT concentrations.
